# Typhoid Fever in Pigs

**Published:** 1865-11

**Authors:** 


TYPHOID FEVER IN PIGS.—Dr. W. Budd states that typhoid fever has destroyed 10,000 to 15,000 pigs in the south-west of England during the last eighteen months.

